# Basic to translational research in dementia: meeting report from the Alzheimer’s Research UK Conference 2014

**DOI:** 10.1186/alzrt270

**Published:** 2014-07-04

**Authors:** Rosa M Sancho, Simon H Ridley, Laura E Phipps, Eric Karran

**Affiliations:** 1 Alzheimer’s Research UK, 3 Riverside, Granta Park, Cambridge CB21 6 AD, UK

## Abstract

This report summarizes the findings presented at the Alzheimer’s Research UK Conference, which was held in Oxford on 25 and 26 March 2014 and which provided an overview of current dementia research from fundamental disease mechanisms to clinical studies.

## Introduction

**Figure 1:**
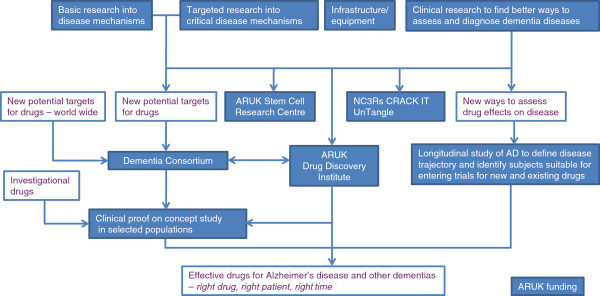
**Alzheimer’s Research UK (ARUK) research strategy to support the delivery of effective dementia treatments.** ARUK funding will continue to support infrastructure and basic to clinical research, and a quarter of this funding will be focused on supporting targeted research into aspects of amyloid-beta biology, tau biology, neuronal death, and mechanisms underpinning the transmission of pathological protein conformers. Funding for the first critical phases of drug discovery will be provided through the Dementia Consortium (http://www.dementiaconsortium.org) and the ARUK Drug Discovery Institute, the latter of which is now being established. A longitudinal study of patients with Alzheimer’s disease (AD) will aim to define early changes and disease trajectory which will eventually help with clinical diagnosis and trial design.

This year the annual Alzheimer’s Research UK (ARUK) Conference returned to Oxford, where it had started in 2000. But what was then a small gathering of a dozen or so ARUK-funded researchers is now the UK’s largest dementia research conference with a varied academic program arranged by the local ARUK Network Centre led by Richard Wade-Martins (University of Oxford, UK). In the meantime, dementia has increasingly been recognized as a societal and economic challenge and research has significantly improved our understanding of this condition. More recently, heightened political interest has led to the G8 Dementia Summit and national initiatives such as the National Alzheimer’s Plan in the US and the Prime Minister’s Challenge on Dementia in the UK. Against this backdrop, Eric Karran outlined ARUK’s efforts to accelerate translational research and drug discovery, as depicted in Figure 1.

## Mechanisms of disease

Evidence supports that the abnormal processing of the amyloid precursor protein (APP) and the formation of amyloid beta (Aβ) trigger a cascade of downstream events in Alzheimer’s disease (AD) which lead to amyloid plaque formation. Jennie Flint (University of Cambridge, UK) presented ongoing work on the detection and characterization of Aβ aggregates by using single-molecule techniques. Flint showed that the predominant oligomers are formed from mixtures of Aβ1-40 and Aβ1-42 with varying rates of aggregation. In response to what lies downstream of Aβ, reports have suggested that interactions between tau and Fyn play a critical role in mediating synaptic toxicity and neuronal loss. Dawn Lau (King’s College London, UK) showed that tau binds to the Src-homology 3 domain of Fyn via the PXXP motifs in tau, an association that may be regulated by phosphorylation. This could affect tau trafficking and extracellular release. The relationship between Aβ and tau in synaptic impairment was explored by Mariana Vargas-Caballero (University of Southampton, UK). Patch clamp studies showed that GluN2B receptor activity was reduced in wild-type hippocampal slices after incubation with Aβ but not in slices from Tau^−/−^ mice, suggesting that tau protein couples Aβ with GluN2B downregulation. Furthermore, expression of H1 MAPT in Tau^−/−^ mice was shown to restore Aβ-mediated inhibition of hippocampal long-term potentiation, whereas expression of mutant N296H showed no effect. However, John H Morrison (Icahn School of Medicine at Mount Sinai, New York, NY, USA) suggested that synaptic plasticity dysfunction could differ between brain regions. Morrison found that in pre-frontal cortex dendrites of non-human primates, highly plastic, N-methyl-D-aspartate receptor-dominated thin spines decreased with age and this loss correlated with cognitive decline. However, Morrison reported minimal synapse loss in the hippocampus and an age-related failure of alpha-amino-3-hydroxy-5-methyl-4-isoxazolepropionic acid receptor insertion in the large stable synapses that correlated with cognitive decline. Synaptic dysfunction results in abnormal patterns of neuronal network activity, as outlined by Jorge J Palop (Gladstone Institute of Neurological Disease and the University of California, San Francisco, CA, USA), whose recent findings suggest that depressed gamma oscillatory rhythmic activity and network hyperexcitability contribute to cognitive decline in hAPPJ20 mice. At a molecular level, Palop and colleagues found decreased levels of voltage-gated sodium channel Nav1.1 subunit (also observed in patients with AD) which resulted in an impairment of inhibitory parvalbumin-expressing interneurons. Interestingly, network and cognitive deficits were restored by enhancing Nav1.1 levels in parvalbumin interneurons.

As neurodegeneration occurs, many processes and pathways are altered. Hugh Perry (University of Southampton, UK) showed that pharmacological inhibition of the colony-stimulating factor 1 receptor and interleukin-34, which drive proliferation and priming of microglia, decreased the numbers of microglia, delayed the onset of behavioral deficits, and prolonged survival in a mouse prion model. Ahmad Khundakar (Newcastle University, UK) presented a series of biochemical and neuropathological studies showing significant changes in synaptic markers underlying glutamate and gamma-aminobutyric acid neurone function in the primary visual cortex in dementia with Lewy bodies (DLB) patients experiencing visual hallucinations compared with AD and control groups. This area, however, was unaffected by α-synuclein, Aβ, or tau pathology. Jose Bras (University College London, UK) further suggested a role for lysosomal dysfunction in the etiology of DLB following an association study of 54 genomic regions previously implicated in Parkinson’s disease (PD) or AD in a large cohort of neuropathologically proven DLB cases and controls. Disease mechanisms in frontotemporal dementia (FTD) were presented by Stuart Pickering-Brown (University of Manchester, UK) and Adrian Isaacs (University College London, UK). An expanded non-coding GGGGCC repeat in C9orf72 is the most common genetic cause of FTD and amyotrophic lateral sclerosis. Pickering-Brown described the generation of antibodies and expression constructs to five di-peptide repeats which recapitulate the p62-, ubiquitin-, and ubiquitin-2-positive inclusions seen in patients. Isaacs showed that both sense and antisense repeat RNAs aggregate in nuclear foci in patient brains and that these aggregates inversely correlated with age at disease onset. Furthermore, Isaacs and colleagues showed that RNA GGGGCC repeats form G-quadruplex structures, which in the future could be targeted by small molecules. These findings were put into context by John R Hodges (FRONTIER Neuroscience Research Australia and the University of New South Wales, Sydney, Australia), who presented current clinical, neuropsychological, and brain imaging research in FTD.

## Biomarker, neuropathological, and clinical studies

Biomarkers for neurodegeneration, particularly those which precede clinical symptoms, hold great promise for the development of accurate diagnostic tests. Simon Lovestone (University of Oxford, UK) argued that efforts to develop an AD blood biomarker should focus on disease groups with detailed clinical, imaging, and molecular datasets rather than the more typical case–control design. Employing antibody and aptamer capture platforms alongside mass spectrometry approaches, Lovestone presented a panel of proteins (including clusterin, DKK proteins, and complement C5) that could predict conversion from mild cognitive impairment to dementia. Zsuzsanna Nagy (University of Birmingham, UK) described the development of a lymphocyte assay that tests the functional integrity of the mammalian target of rapamycin (mTOR) pathway in AD. Nagy suggested that, rather than changes in a single gene, dysregulation of the mTOR pathway as a whole could serve as an accurate risk measure for neurodegeneration. Clare Mackay (University of Oxford, UK) proposed the use of ‘at-risk’ groups to develop imaging biomarkers. Using resting functional magnetic resonance imaging (fMRI), Mackay showed that reductions in resting connectivity of the default mode network in AD are mirrored in young healthy apolipoprotein E4 allele carriers. In parallel work, reductions in basal ganglia connectivity in patients with PD were also detected in people at increased risk of PD by virtue of having rapid eye movement sleep disorder or being asymptomatic mutation carriers. Ruth Cromarty (Newcastle University, UK) suggested that distributed attention-executive network inefficiencies occur in both DLB and AD although no specific area of the network was particularly impaired. Hannah Golden (University College London, UK) used auditory scene analysis to show deficits in spatial processing, auditory motion detection, and stationary position discrimination of sound in patients with AD and posterior cortical atrophy. These deficits were associated with the right inferior parietal lobe, a region associated with the default mode network. James Rowe (University of Cambridge, UK) discussed techniques to correlate and model information from diffusion tensor imaging (DTI), fMRI, and magnetoencephalography. In addition to elucidating network connectivity and efficiency in cognitive decline, these methods have the potential to improve sensitivity for diagnosis and treatment evaluation. This topic was further explored by Hugh Markus (University of Cambridge, UK), who showed recent advances in the detection of disrupted networks in patients with small vessel disease by using DTI and graph theory. The changes were distinct from AD in that they correlate with cognitive domains such as executive function and information-processing speed, which are particularly affected at early stages of the disease. Markus highlighted that such differences have allowed the development of screening tests, such as the Brief Memory and Executive Test, that are more sensitive than the Mini-Mental State Examination. The clinical and pathological overlap between AD and vascular dementia was further discussed by Raj Kalaria (Newcastle University, UK). Kalaria suggested that vascular disease risk is strongly associated with late-onset AD and that vascular factors affect the frontal lobe but that neurodegenerative pathologies propagate largely from the temporal lobe, operating simultaneously through metabolic alterations or other processes to cause dementia.

## Prevention and therapy development

Wade-Martins and Carol Brayne (University of Cambridge, UK) chaired a lively debate on the relative importance of genes and environment in dementia. John Gallacher (Cardiff University, UK) and Karen Ritchie (Imperial College London, UK, and INSERM, France) argued that modifiable factors have a substantial impact on dementia incidence, as evidenced by epidemiological studies such as the Honolulu-Asia Aging Study, CAIDE (Cardiovascular Risk Factors, Aging, and Dementia), or the Caerphilly study. On a related topic, Cheryl Hawkes (University of Southampton, UK) had shown that murine maternal obesity during gestation and lactation, as well as sustained high-fat diet throughout life, altered inflammatory and basement membrane factors and impaired perivascular clearance of Aβ from the mouse brain. In contrast, Kevin Morgan (University of Nottingham, UK) and Julie Williams (Cardiff University, UK) defended the importance of genetics in identifying new disease mechanisms, such as cholesterol metabolism, immune function, and synaptic vesicle recycling. They also discussed the identification of increased risk variants, such as *TREM2* and *PLD3*, arguing that these findings are crucial to the development of preventative and therapeutic measures. The debate ended with an agreement that collaboration and a shared appreciation of both fields were essential to improve understanding.

A keynote presentation by Chas Bountra (University of Oxford, UK) highlighted the work being carried out by the Structural Genomics Consortium which focuses on kinases and epigenetics as potential drug targets for neurodegenerative diseases and screening novel inhibitors on patient-derived cells. An emphasis was put on the need for open collaborations between academia and industry, resource and risk sharing, and active participation of patient organizations and regulators. Lovestone further proposed the development of adaptive clinical trials on existing cohorts with well-characterized clinical and biomarker data, in which treatment regimens are adjusted depending on amyloid burden and cognition changes.

In conclusion, the ARUK Conference in Oxford highlighted a range of approaches to understand the causes and progression of dementia, and we hope you will join us next year in London on 10 and 11 March 2015.

## Abbreviations

AD: Alzheimer’s disease; APP: Amyloid precursor protein; ARUK: Alzheimer’s Research UK; Aβ: Amyloid beta; DLB: Dementia with Lewy bodies; DTI: Diffusion tensor imaging; fMRI: Functional magnetic resonance imaging; FTD: Frontotemporal dementia; mTOR: Mammalian target of rapamycin; PD: Parkinson’s disease.

## Competing interests

The authors declare that they have no competing interests.

